# F. H. Davis, M.D.

**Published:** 1880-12

**Authors:** 


### Obituary.

---

REPORT OF MEMORIAL COMMITTEE, CHICAGO MEDICAL SOCIETY, IN VIEW OF THE DECEASE OF THE LATE DR. F. H. DAVIS, PRESENTED NOV. 15, 1880.

Dr. Frank Howard Davis was born in the city of New York in 1848. He began the study of medicine in 1867, at the Chicago Medical College, and was graduated in 1871. At the same college he received the prize for the best thesis presented to the faculty by the class of that year.

In order to prepare himself more thoroughly for the duties of his chosen profession, he spent several years abroad, dividing his time between London, Paris and Vienna, and devoting himself specially to the study and practice of laryngoscopy. That he was studious and active in his profession is evident from the number of appointments with which he was favored. He was treasurer of the Chicago Medical Society. He was secretary of the Association of American Editors; also secretary of the American Laryngological Association. He was an active member of the American Medical Association, the Illinois State Medical Society and the Alumni Association of the Chicago Medical College. He was also an active member of the Chicago Academy of Sciences, of which he was librarian at the time of his death.

The doctor wrote a number of articles, which have appeared in different medical journals, and which have also been published in the transactions of the associations with which he was connected. Among the more important were probably his inaugural thesis: "Atmospheric Germs and their Relation to Disease" (published in *Chicago Medical Examiner*, vol. 12, number 4). "Some

Facts regarding the Relations existing between the Prevalence of Pulmonary Tuberculosis and Hygienic Surroundings of the People" (American Medical Association Transactions 1878, p. 147). "A Study of Nine Hundred and Sixty-five Cases of Chronic Pulmonary Disease" (American Med. Ass'n 1877, p. 269). "Clinical Lectures on Various Important Diseases," by Dr. N. S. Davis; edited by Frank H. Davis (published by H. C. Lea, Phila.). "Inhalations in Pulmonary Diseases" (Illinois Medical Society, 1880).

The doctor in 1875 was united in marriage to Miss Anna Smith Marcy, daughter of Prof. Oliver Marcy, of the North-western University.

Their marriage was blessed with two children—a son and a daughter, who, with their mother, now mourn the loss of a most devoted husband and affectionate father. Few young men had the opportunities of preparing themselves for the arduous and responsible duties of life that the doctor enjoyed, and no one ever employed them to greater advantage. The chief cause of his death was "suppurative inflammation of the left kidney," accompanied by so persistent a reflex irritation of the stomach that during the last few weeks of life his sole dependence for support was on nutritive enemas. The doctor had the highest regard for professional honor, but those only who knew him intimately could appreciate fully his kind, sensitive nature.

WHEREAS, The Chicago Medical Society has heard with great regret of the death of Frank Howard Davis, long a member and officer of this society,

*Resolved*, That the Chicago Medical Society is called upon to mourn the loss of an active, efficient member and officer, who was ever faithful in the discharge of his duties.

*Resolved*, That as a tribute of respect to his memory, we wish to place upon our records our appreciation of his character and of his services to the society and the profession.

*Resolved*, That while we fully realize our own loss, we would extend our most heartfelt sympathy and condolence to his afflicted family, and particularly his bereaved father, to whom we are all personally greatly attached.

*Resolved*, That a copy of these resolutions of respect and condolence be sent to his family, and also to the city medical journals and daily press for publication.

Your committee,

H. P. MERRIMAN,
LYMAN WARE,
ROSWELL PARK.

Per L. H. MONTGOMERY,
*Secretary Chicago Medical Society.*

